# Supplementary material for: Predicting the host of influenza viruses based on the word vector
Source: PeerJ. 2017 Jul 18;5:e3579. doi: 10.7717/peerj.3579 (PMC5518728; doi:10.7717/peerj.3579)
Supplement: Supplemental Information 1 [file peerj-05-3579-s001.docx]

**Supplementary information for**

**Predicting the host of influenza viruses based on the word vector**

Beibei Xu^1^, Zhiying Tan^1^, Kenli Li^1^, Taijiao Jiang^2, 3, *^, Yousong Peng^4,*^

^1^ College of Computer Science and Electronic Engineering, Hunan University, Changsha, 410082, China

^2^ Center of System Medicine, Institute of Basic Medical Sciences, Chinese Academy of Medical Sciences & Peking Union Medical College, Beijing, 100005, China

^3^ Suzhou Institute of Systems Medicine, Suzhou, Jiangsu, 215123, China

^4^ College of Biology, Hunan University, Changsha, 410082, China

* To whom correspondence should be addressed. Email: [taijiao@moon.ibp.ac.cn](mailto:taijiao@moon.ibp.ac.cn)(TJ) or [pys2013@hnu.edu.cn](mailto:pys2013@hnu.edu.cn)(YP)

**Supplementary Figures and Tables**

**Table S1** The number of non-redundant DNA sequences used in this study for each segment of avian, human and swine influenza A viruses

| Genes | Avian | Human | Swine |
| --- | --- | --- | --- |
| HA | 12937 | 22453 | 5124 |
| NA | 9746 | 13207 | 3191 |
| PB2 | 8835 | 7241 | 1567 |
| PB1 | 8600 | 7057 | 1596 |
| PA | 8570 | 6890 | 1554 |
| NP | 7731 | 5963 | 1589 |
| NS | 7261 | 4735 | 1428 |
| MP | 7484 | 6598 | 2309 |

**Table S2** Performances for models based on the word vector with words of two to four amino acids long derived from the influenza protein dataset. The accuracies are averaged in ten-fold cross-validations.

| Protein | Host | The length of word vectors | | |
| --- | --- | --- | --- | --- |
|  |  | Two | Three | Four |
| HA | Avian | 0.996 | 0.997 | 0.997 |
|  | Human | 0.910 | 0.937 | 0.959 |
|  | Swine | 0.628 | 0.774 | 0.905 |
|  | Overall | 0.893 | 0.931 | 0.964 |
| NA | Avian | 0.993 | 0.992 | 0.995 |
|  | Human | 0.883 | 0.898 | 0.944 |
|  | Swine | 0.762 | 0.759 | 0.894 |
|  | Overall | 0.905 | 0.910 | 0.955 |
| PB2 | Avian | 0.993 | 0.995 | 0.993 |
|  | Human | 0.916 | 0.874 | 0.903 |
|  | Swine | 0.752 | 0.621 | 0.769 |
|  | Overall | 0.932 | 0.900 | 0.931 |
| PB1 | Avian | 0.991 | 0.993 | 0.993 |
|  | Human | 0.911 | 0.867 | 0.902 |
|  | Swine | 0.775 | 0.676 | 0.773 |
|  | Overall | 0.930 | 0.902 | 0.928 |
| PA | Avian | 0.993 | 0.995 | 0.993 |
|  | Human | 0.896 | 0.843 | 0.895 |
|  | Swine | 0.753 | 0.617 | 0.801 |
|  | Overall | 0.925 | 0.887 | 0.933 |
| NP | Avian | 0.988 | 0.988 | 0.989 |
|  | Human | 0.790 | 0.664 | 0.842 |
|  | Swine | 0.704 | 0.421 | 0.802 |
|  | Overall | 0.878 | 0.785 | 0.912 |
| M1 | Avian | 0.978 | 0.972 | 0.978 |
|  | Human | 0.584 | 0.394 | 0.577 |
|  | Swine | 0.383 | 0.151 | 0.411 |
|  | Overall | 0.707 | 0.588 | 0.712 |
| M2 | Avian | 0.969 | 0.970 | 0.970 |
|  | Human | 0.111 | 0.012 | 0.205 |
|  | Swine | 0.022 | 0.001 | 0.057 |
|  | Overall | 0.472 | 0.440 | 0.509 |
| NS1 | Avian | 0.987 | 0.989 | 0.987 |
|  | Human | 0.455 | 0.402 | 0.765 |
|  | Swine | 0.134 | 0.055 | 0.364 |
|  | Overall | 0.662 | 0.632 | 0.799 |
| NS2 | Avian | 0.968 | 0.966 | 0.968 |
|  | Human | 0.037 | 0.007 | 0.145 |
|  | Swine | 0.011 | 0.000 | 0.064 |
|  | Overall | 0.522 | 0.512 | 0.561 |
| PB1-F2 | Avian | 0.990 | 0.992 | 0.989 |
|  | Human | 0.208 | 0.024 | 0.249 |
|  | Swine | 0.057 | 0.001 | 0.070 |
|  | Overall | 0.703 | 0.662 | 0.712 |
| PA-X | Avian | 0.992 | 0.993 | 0.992 |
|  | Human | 0.305 | 0.028 | 0.295 |
|  | Swine | 0.197 | 0.009 | 0.205 |
|  | Overall | 0.625 | 0.513 | 0.625 |

**Table S3** Performances for models based on the word vector with words of two to four amino acids long derived from the SwissProt dataset. The accuracies are averaged in ten-fold cross-validations.

| Protein | Host | The length of word vectors | | |
| --- | --- | --- | --- | --- |
|  |  | Two | Three | Four |
| HA | Avian | 0.997 | 0.997 | 0.997 |
|  | Human | 0.947 | 0.934 | 0.907 |
|  | Swine | 0.795 | 0.707 | 0.677 |
|  | Overall | 0.939 | 0.918 | 0.900 |
| NA | Avian | 0.994 | 0.992 | 0.993 |
|  | Human | 0.910 | 0.894 | 0.884 |
|  | Swine | 0.870 | 0.817 | 0.757 |
|  | Overall | 0.937 | 0.919 | 0.904 |
| PB2 | Avian | 0.995 | 0.995 | 0.994 |
|  | Human | 0.912 | 0.912 | 0.913 |
|  | Swine | 0.773 | 0.788 | 0.776 |
|  | Overall | 0.935 | 0.938 | 0.935 |
| PB1 | Avian | 0.993 | 0.995 | 0.992 |
|  | Human | 0.900 | 0.906 | 0.909 |
|  | Swine | 0.775 | 0.781 | 0.781 |
|  | Overall | 0.928 | 0.932 | 0.931 |
| PA | Avian | 0.993 | 0.992 | 0.993 |
|  | Human | 0.900 | 0.905 | 0.898 |
|  | Swine | 0.794 | 0.794 | 0.788 |
|  | Overall | 0.932 | 0.934 | 0.931 |
| NP | Avian | 0.989 | 0.990 | 0.989 |
|  | Human | 0.856 | 0.833 | 0.816 |
|  | Swine | 0.852 | 0.792 | 0.737 |
|  | Overall | 0.926 | 0.908 | 0.891 |
| M1 | Avian | 0.983 | 0.979 | 0.975 |
|  | Human | 0.704 | 0.712 | 0.586 |
|  | Swine | 0.609 | 0.543 | 0.351 |
|  | Overall | 0.803 | 0.786 | 0.698 |
| M2 | Avian | 0.969 | 0.967 | 0.968 |
|  | Human | 0.107 | 0.012 | 0.021 |
|  | Swine | 0.020 | 0.008 | 0.006 |
|  | Overall | 0.471 | 0.440 | 0.443 |
| NS1 | Avian | 0.987 | 0.989 | 0.988 |
|  | Human | 0.654 | 0.452 | 0.468 |
|  | Swine | 0.238 | 0.084 | 0.108 |
|  | Overall | 0.741 | 0.652 | 0.662 |
| NS2 | Avian | 0.968 | 0.967 | 0.968 |
|  | Human | 0.011 | 0.007 | 0.009 |
|  | Swine | 0.008 | 0.006 | 0.001 |
|  | Overall | 0.516 | 0.513 | 0.513 |
| PB1-F2 | Avian | 0.989 | 0.990 | 0.990 |
|  | Human | 0.324 | 0.111 | 0.019 |
|  | Swine | 0.065 | 0.012 | 0.004 |
|  | Overall | 0.726 | 0.679 | 0.660 |
| PA-X | Avian | 0.994 | 0.992 | 0.994 |
|  | Human | 0.562 | 0.348 | 0.260 |
|  | Swine | 0.549 | 0.267 | 0.174 |
|  | Overall | 0.778 | 0.653 | 0.609 |

**Table S4** Performances for models based on the word vector with words of two to four nucleotides long derived from the influenza DNA dataset. The accuracies are averaged in ten-fold cross-validations.

| Gene | Host | The length of word vectors | | |
| --- | --- | --- | --- | --- |
|  |  | Two | Three | Four |
| HA | Avian | 0.980 | 0.997 | 0.997 |
|  | Human | 0.967 | 0.969 | 0.957 |
|  | Swine | 0.800 | 0.906 | 0.626 |
|  | Whole | 0.950 | 0.970 | 0.928 |
| NA | Avian | 0.986 | 0.995 | 0.995 |
|  | Human | 0.955 | 0.958 | 0.948 |
|  | Swine | 0.694 | 0.897 | 0.705 |
|  | Whole | 0.934 | 0.964 | 0.935 |
| PB2 | Avian | 0.826 | 0.990 | 0.995 |
|  | Human | 0.941 | 0.954 | 0.951 |
|  | Swine | 0.717 | 0.796 | 0.828 |
|  | Whole | 0.864 | 0.958 | 0.962 |
| PB1 | Avian | 0.971 | 0.987 | 0.995 |
|  | Human | 0.931 | 0.950 | 0.949 |
|  | Swine | 0.597 | 0.811 | 0.830 |
|  | Whole | 0.920 | 0.955 | 0.961 |
| PA | Avian | 0.912 | 0.993 | 0.994 |
|  | Human | 0.946 | 0.953 | 0.945 |
|  | Swine | 0.313 | 0.793 | 0.820 |
|  | Whole | 0.871 | 0.959 | 0.959 |
| NP | Avian | 0.975 | 0.994 | 0.994 |
|  | Human | 0.931 | 0.938 | 0.944 |
|  | Swine | 0.662 | 0.870 | 0.736 |
|  | Whole | 0.925 | 0.959 | 0.948 |
| MP | Avian | 0.982 | 0.995 | 0.997 |
|  | Human | 0.928 | 0.931 | 0.890 |
|  | Swine | 0.475 | 0.751 | 0.544 |
|  | Whole | 0.889 | 0.935 | 0.890 |
| NS | Avian | 0.994 | 0.998 | 0.998 |
|  | Human | 0.894 | 0.800 | 0.639 |
|  | Swine | 0.539 | 0.174 | 0.104 |
|  | Whole | 0.910 | 0.840 | 0.777 |

**Table S5** Comparison of methods for predicting host of influenza A viruses based on word vectors and sequence homology best hit search using the influenza DNA sequences. It listed the overall accuracies for predicting the host of influenza A viruses. For the former, the best of its kind was used.

| Gene | Methods for predicting host of influenza A virus | | |
| --- | --- | --- | --- |
|  | Word Vector | BLAST | HMM |
| HA | 0.970 | 0.979 | 0.779 |
| NA | 0.964 | 0.972 | 0.599 |
| PB2 | 0.958 | 0.974 | 0.797 |
| PB1 | 0.955 | 0.974 | 0.941 |
| PA | 0.959 | 0.945 | 0.885 |
| NP | 0.964 | 0.967 | 0.813 |
| MP | 0.935 | 0.959 | 0.869 |
| NS | 0.840 | 0.957 | 0.844 |
